# Pediatric hypophosphatasia: lessons learned from a retrospective single-center chart review of 50 children

**DOI:** 10.1186/s13023-020-01500-x

**Published:** 2020-08-18

**Authors:** Marius Vogt, Hermann Girschick, Tilmann Schweitzer, Clemens Benoit, Annette Holl-Wieden, Lothar Seefried, Franz Jakob, Christine Hofmann

**Affiliations:** 1 Pediatric Rheumatology and Osteology, University Children’s Hospital Wuerzburg, Wuerzburg, Germany; 2 Children’s Hospital, Vivantes Hospital im Friedrichshain, Berlin, Germany; 3 Department of Neurosurgery, Section of Pediatric Neurosurgery, University Hospital of Würzburg, Wuerzburg, Germany; 4 Institute of Radiology, Division of Pediatric Radiology, University Hospital of Würzburg, Wuerzburg, Germany; 5 Bernhard-Heine-Center for Locomotion Research, Orthopedic Department, University of Wuerzburg, Wuerzburg, Germany

**Keywords:** Hypophosphatasia, Alkaline phosphatase, Asfotase alfa, Rare bone disease, Osteomalacia, Rickets

## Abstract

**Background:**

Hypophosphatasia (HPP) is a rare, inherited metabolic disorder caused by loss-of-function mutations in the ALPL gene that encodes the tissue-nonspecific alkaline phosphatase TNAP (ORPHA 436). Its clinical presentation is highly heterogeneous with a remarkably wide-ranging severity. HPP affects patients of all ages. In children HPP-related musculoskeletal symptoms may mimic rheumatologic conditions and diagnosis is often difficult and delayed. To improve the understanding of HPP in children and in order to shorten the diagnostic time span in the future we studied the natural history of the disease in our large cohort of pediatric patients.

This single centre retrospective chart review included longitudinal data from 50 patients with HPP diagnosed and followed at the University Children’s Hospital Wuerzburg, Germany over the last 25 years.

**Results:**

The cohort comprises 4 (8%) perinatal, 17 (34%) infantile and 29 (58%) childhood onset HPP patients. Two patients were deceased at the time of data collection. Diagnosis was based on available characteristic clinical symptoms (in 88%), low alkaline phosphatase (AP) activity (in 96%), accumulating substrates of AP (in 58%) and X-ray findings (in 48%). Genetic analysis was performed in 48 patients (31 compound heterozygous, 15 heterozygous, 2 homozygous mutations per patient), allowing investigations on genotype-phenotype correlations. Based on anamnestic data, median age at first clinical symptoms was 3.5 months (min. 0, max. 107), while median time to diagnosis was 13 months (min. 0, max. 103). Common symptoms included: impairment of motor skills (78%), impairment of mineralization (72%), premature loss of teeth (64%), musculoskeletal pain and craniosynostosis (each 64%) and failure to thrive (62%). Up to now 20 patients started medical treatment with Asfotase alfa.

**Conclusions:**

Reported findings support the clinical perception of HPP being a chronic multi-systemic disease with often delayed diagnosis. Our natural history information provides detailed insights into the prevalence of different symptoms, which can help to improve and shorten diagnostics and thereby lead to an optimised medical care, especially with promising therapeutic options such as enzyme-replacement-therapy with Asfotase alfa in mind.

## Background

Hypophosphatasie (HPP) is a rare, inherited, systemic, metabolic disorder that was first described by J.C. Rathbun in 1948 (ORPHA 436) [1]. It is caused by loss-of-function-mutations in the ALPL gene, encoding tissue-nonspecific alkaline phosphatase (TNAP). It results in reduced serum alkaline phosphatase (AP) activity and increased extracellular accumulation of TNAP substrates such as inorganic pyrophosphate (PPi), pyridoxal-5′-phosphate (PLP, the circulating form of vitamin B6) or phosphoethanolamine (PEA) [2]. PPi is a known inhibitor of hydroxyapatite crystal formation and propagation and therefore acts as a potent calcification inhibitor [3]. Furthermore, it supposedly supports the deposition of pyrophosphate and calcium outside the osteoblasts in a crystallised form, which - similar to PPi itself – may cause inflammatory processes such as arthritis or chronic non-bacterial inflammation [3]. PLP is an essential cofactor in many biochemical processes of the human body, e.g. in the synthesis of important neurotransmitters such as gamma aminobutyric acid (GABA), serotonin or dopamine, and must be dephosphorylated to pyridoxal (PL) to be able to pass the blood-brain barrier as well as cell membranes. This process is catalysed by TNAP [4]. There are only a few studies that address HPP incidence and prevalence. Life-threatening HPP occurs in approximately 1 per 100,000 and 300,000 births in Canada and in Europe, respectively [5]. Due to low selective pressure and a large number of undiagnosed patients, the prevalence of mild forms of HPP is suspected to be much higher. Based on ALPL gene mutation analysis the prevalence in the European population was estimated to be 1/16370 [6].

Patients with HPP show a wide range of different symptoms, even among individuals with the same genotype or in the same family, so elaborating HPP as the primary disease is a great challenge for attending physicians. Based on clinical characteristics and the age of onset HPP has been classified in five different subtypes: perinatal (severe symptoms in utero or within the first 4 weeks of life), infantile (symptoms start after 4 weeks but before 6 months of life), childhood (first symptoms after first 6 months of life), adult (first symptoms in adulthood), odonto HPP [7–10]. The characteristic manifestations of HPP in infants may include failure to thrive, rickets-like deformities, pulmonary insufficiency, muscle weakness, nephrocalcinosis, premature craniosynostosis, and vitamin B6-responsive seizures; in toddlers, young children, and adolescents, premature tooth loss, bone deformities and rachitic changes in long bones, musculoskeletal pain and delayed motor development are characteristic [2, 11]. In particular children and adolescents with predominant musculoskeletal problems may apply to a for example pediatric rheumatology outpatient clinic as they are often misdiagnosed as for example pain amplification syndrome, chronic non-bacterial osteomyelitis or diffuse chronic arthralgia/myalgia [3, 7, 8].

The natural history of HPP is poorly understood at this time, likely because of the rarity and wide clinical heterogeneity of the disease. Early onset forms (perinatal, infantile) of HPP show a high mortality, while other forms seem to go along with normal life expectancy but can be associated with significant burden of disease, including but not limited to functional disability due to musculoskeletal problems as well as reduced physical activity and quality of life.

Clinical management and treatment of HPP is based on supportive measures that address the symptoms of the disease (e.g., respiratory support, nutrition, orthopedic or neurosurgical intervention, pain relief) [12]. In particular young children and adolescents with pain in their lower limbs and inflammatory lesions in the MRI seem to profit from an anti-inflammatory therapy with NSAIDs [12]. Since 2015 Asfotase alfa (AA, Strensiq®, Alexion Pharmaceuticals, Inc., Boston, MA, USA), a human recombinant TNAP enzyme replacement therapy, is approved in many countries for long-term enzyme replacement therapy (ERT) in patients with pediatric-onset HPP to treat bone manifestations of the disease [13, 14]. Treatment with AA was associated with skeletal, respiratory and functional improvement and was considered a milestone in the management of severe forms with improved overall survival in this cohort [15–18]. But also young children, and adolescents affected by milder forms of HPP have been shown to benefit from AA by improving physical activity and reducing pain [19]. No data is available on potentially therapeutic modification of neurological, gastrointestinal and cardiovascular problems of HPP patients treated with AA.

HPP has a very heterogeneous presentation, which, coupled with its rarity, often leads to missed or delayed diagnosis and an incomplete understanding of its natural history. To better understand the epidemiology and clinical course of pediatric HPP, including first clinical symptoms, timing of diagnosis, genotype phenotype correlation of the disease we present data of our German referral center retrospective chart review. Especially in the light of an effective bone targeted treatment it seems to be important to improve our understanding of pediatric HPP to shorten the diagnostic time span and thereby enable an optimised medical care and to avoid or postpone disease-related symptoms and complications.

## Methods

We performed a retrospective chart review, which included longitudinal demographic and clinical data from all pediatric patients who had been diagnosed and followed at the University Children’s Hospital (Section of Pediatric Rheumatology and Osteology) in Wuerzburg over the last 25 years. The center has a great history and experience with this disease and the German patients´ organisation is also based in this city. Eligibility criteria were the presentation of characteristic clinical symptoms associated with HPP in addition to a reduced AP activity (accordimg to age and gender adjusted normal values) in serum and/or at least one documented mutation in the ALPL gene. In total, we identified 50 patients with appropriate documentation to confirm these criteria. For each patient we collected data concerning age and gender, clinical subtype, age at onset, first HPP-related symptoms, age at diagnosis, findings leading to the HPP diagnosis, previous misdiagnoses or differential diagnoses, laboratory findings (AP, PLP, PEA, vitamin D, calcium, phosphate), genetic findings and treatment in a Microsoft Access® database.

## Results

### Demographics

Our cohort of 50 HPP patients (26 male, 24 female) consists of 4 perinatal (8%), 17 infantile (34%) and 29 childhood (58%) onset HPP patients. Up to now, two patients (both of perinatal subtype) died at the age of 3 months, due to cardiac arrest and deleterious neurological outcome [15].

### HPP medical history

Median age at first HPP symptoms was 3.5 (min. 0, max. 107) months, median age at HPP diagnosis was 24 (min. 0, max. 127) months, and median time to diagnosis was 13 (min. 0, max. 103) months (Table 1). There was no delay in making the diagnosis in perinatal HPP. Median time from the first attributable symptom to diagnosis in infantile HPP was 12 months, in childhood HPP 22.5 months.

**Table 1:** Median age at first symptoms, diagnosis and median time to diagnosis

*Age (in months)*	Perinatal HPP	Infantile HPP	Childhood HPP	All Subtypes
Median age at first symptoms	(min. 0; max. 0)	2 (0; 14)	9 (0; 107)	3.5 (0; 107)
Median age at diagnosis	0 (0; 0)	15 (2; 49)	36 (3; 127)	24 (0; 127)
Median time to diagnosis	0 (0; 0)	12 (1; 45)	22.5 (0; 103)	13 (0; 103)
		♂: 12 (2; 24)	♂: 21 (0; 86)	♂: 13 (0; 103)
		♀: 9 (1; 45)	♀: 22.5 (0; 103)	♀: 15.5 (0; 86)

In 82.1% (32/39) of patients with sufficiently documented data, the diagnosis was made in hospital, 7.7% (3/39) of the patients were diagnosed by pediatric practitioners, 7.7% (3/39) by dentists and one patient by a general practitioner.

The most frequent first symptoms in our cohort were failure to thrive in 34% (17/50) followed by an abnormally shaped head / prominent fontanel in 22% (11/50) and a premature loss of teeth in 20% (10/50) of the patients. Less frequent first symptoms were: nutritional problems (14%, 7/50), respiratory problems (12%, 5/50), muscle weakness (12%, 6/50), bone deformities (8%, 4/50), abnormally shaped thorax (8%, 4/50), impairment of motor skills (4%, 2/50), parodontitis (2%, 1/50), musculoskeletal pain (2%, 1/50), cerebral seizures (2%, 1/50). In some patients a composite of symptoms was noticed from the beginning.

In 21 patients (42%), at least one differential diagnosis was documented before diagnosing HPP as the underlying cause of the presented symptoms. Ten patients were diagnosed with various forms of rickets and based on a failure to thrive, two patients were each suspected with cystic fibrosis and a lack of growth hormone. Further documented differential diagnoses were hypo−/achondroplasia, different forms of food intolerance, neurofibromatosis, polyarthritis, tethered-chord syndrome, chronic non-bacterial osteomyelitis, Langerhans cell histiocytosis or extradermal dysplasia (one each).

During the course of disease, the patients in our cohort presented a wide range of different symptoms. We divided these symptoms into various categories (Table 2). All patients with perinatal HPP showed pulmonary abnormalities, cerebral seizures and impairment of mineralization. In addition, 3 out of 4 patients with perinatal HPP had early impairment of motor skills, gastrointestinal problems and craniosynostosis. In infantile HPP most frequently reported findings were: impairment of motor skills (94%), impairment of mineralization (88%), nephrocalcinosis and craniosynostosis (76% each), failure to thrive (71%), pulmonary abnormalities (65%) and premature loss of deciduous teeth (59%). Patients with childhood HPP presented with impairment of motor skills, premature loss of deciduous teeth (69% each), impairment of mineralization (62%) and failure to thrive (59%). Looking at our cohort of 50 patients in total impairment of motor skills (78%), impairment of mineralization (72%) and premature loss of deciduous teeth (64%) were most commonly reported in the HPP-related disease history. Pathologic fractures were found in 14% of the patients.

**Table 2:** HPP-related disease history in different subtypes of HPP

	Perinatal HPP	Infantile HPP	Childhood HPP	Total
	(*n* = 4)	(*n* = 17)	(*n* = 29)	(*n* = 50)
**Systemic manifestations**				
Impairment of motor skills	3/4 (75%)	16/17 (94%)	20/29 (69%)	39/50 (78%)
Speech developmental delay	2/4 (50%)	3/17 (18%)	5/29 (17%)	10/50 (20%)
Failure to thrive	2/4 (50%)	12/17 (71%)	17/29 (59%)	31/50 (62%)
Pulmonary abnormalities	4/4 (100%)	11/17 (65%)	5/29 (17%)	20/50 (40%)
Cardiac abnormalities	2/4 (50%)	5/17 (29%)	6/29 (21%)	13/50 (26%)
Nephrocalcinosis	2/4 (50%)	13/17 (76%)	5/29 (17%)	19/50 (38%)
Cerebral seizures	4/4 (100%)	0/17 (0%)	0/29 (0%)	4/50 (8%)
Gastrointestinal abnormalities	3/4 (75%)	7/17 (41%)	6/29 (21%)	16/50 (32%)
Musculoskeletal pain	1/4 (25%)	7/17 (41%)	16/29 (55%)	24/50 (48%)
Difficulties in swallowing	2/4 (50%)	7/17 (41%)	8/29 (28%)	17/50 (34%)
**Osseous manifestations**				
Impairment of mineralization	4/4 (100%)	15/17 (88%)	18/29 (62%)	36/50 (72%)
Pathologic fractures	1/4 (25%)	4/17 (24%)	1/29 (3%)	6/50 (12%)
Chronic non-bacterial osteomyelitis/osseous inflammation	0/4 (0%)	4/17 (24%)	3/29 (10%)	7/50 (14%)
Craniosynostosis	3/4 (75%)	13/17 (76%)	8/29 (28%)	24/50 (48%)
**Dental manifestations**				
Premature loss-of-teeth	2/4 (50%)	10/17 (59%)	20/29 (69%)	32/50 (64%)
Caries/deficient enamel	1/4 (25%)	2/17 (12%)	7/29 (24%)	10/50 (20%)

### Laboratory findings

All patients showed a reduced activity of AP in laboratory blood testing at least once. In patients with perinatal HPP there was almost no detectable residual AP activity (median 0.5 U/l). Elevated calcium levels (median 2.82 mmol/l, [normal range: 2.0–2.7]) were solely found in the perinatal subtype, while phosphate levels were noted in the upper normal range of all subtypes. Median vitamin D (25 (OH) vitamin D) was reduced in patients with childhood HPP, but in the lower normal range for perinatal and infantile HPP. Parathyroid hormone (PTH) was found to be in the lower normal range. PLP was considerably elevated (median 142.5 ng/ml [5–30]). All our patients with perinatal HPP were supplemented with pyridoxin due to cerebral seizures, thus measuring PLP was not diagnostically meaningful since lab test for pyridoxal phosphate do not discriminate these two forms properly (Table 3).

**Table 3:** Laboratory findings

	Perinatal HPP			Infantile HPP			Childhood HPP			All subtypes		
	Median	Min	Max	Median	Min	Max	Median	Min	Max	Median	Min	Max
AP	0.5	0	18	30	20	77	45	19	139	31.5	0	139
(in U/l)	(*n* = 4)			(*n* = 17)			(*n* = 29)			(*n* = 50)		
Calcium	2.82	2.5	3.6	2.5	2.25	3.72	2.48	2.2	2.8	2.5	2.2	3.72
(in mmol/l), [norm: 2.0–2.7]	(*n* = 4)			(*n* = 17)			(*n* = 28)			(*n* = 49)		
Phosphate	1.91	1.09	2.52	2.02	1.26	2.36	2.03	1.25	2.64	2.02	1.09	2.64
(in mmol/l), [0.97–2.2]	(*n* = 4)			(*n* = 17)			(*n* = 28)			(*n* = 49)		
25(OH)D	33.8	12.6	39	30.8	8.3	50.7	23.6	13.8	53	26.1	8.3	53
(µg/l), [30–70]	(*n* = 3)			(*n* = 15)			(*n* = 21)			(*n* = 39)		
PTH	21.4	7	175	5.3	2	33.6	13.95	3	49	12.3	2	175
(in ng/l), [12–65]	(*n* = 3)			(n = 17)			(*n* = 24)			(*n* = 44)		
PLP	substituted			150	62	774	120	30	446	142.5	30	774
(in ng/ml), [5–30]				(*n* = 11)			(*n* = 19)			(*n* = 30)		

all diagnostic reference levels according to intern laboratory standards

*AP* alkaline phosphatase, *25(OH)D* 25-OH-Vitamin D, *PTH* parathyroid hormone, *PLP* pyridoxal phosphate; * reference levels of AP (37 C°, IFCC method): infants 110–590 IU/l, toddler 110–550 IU/l, pupil 130–700 IU/l according to local laboratory standards)

### Genetics

Genetic testing was performed and sufficiently documented in 48 patients (Table 4). In our cohort, 31 (64.5%) patients are compound-heterozygous, 15 (31.3%) are heterozygous (with 2 being listed as dominant negative mutations [20]) and 2 are homozygous for mutations in the ALPL gene. There are 83 documented mutations in total, of which 80 mutations are located in exons and 3 mutations in introns of the ALPL-gene. As some mutations occur more frequently than others, we found 37 different mutations in our cohort, of which 35 are listed in the online ALPL gene mutation database by E. Mornet [20]. Two mutations have not been published yet but differ only slightly from listed mutations (p.Tyr236*, c.708 T > G and p.Ala443Gly, c.1328C > G). Seventy-six mutations were missense mutations, 4 small deletions/insertions and 4 were stop-mutations.

**Table 4:** Mutations of the ALPL gene found in our cohort

phenotype of the patient	mutation 1	mutation 2	genotype of the patient
perinatal	p.Gln32* (c.94C > T)	c.648 + 1G > A	compound heterozygous
perinatal	p.Arg223Trp (c.667C > T)	p.Arg391Cys (c.1171C > T)	compound heterozygous
perinatal	p.Arg223Trp (c.667C > T)	p.Tyr441* (c.1323C > A)	compound heterozygous
perinatal	p.Ala433Gly (c.1328C > G)	p.Ala433Gly (c.1328C > G)	homozygous
infantile	p.Ala33Val (c.98C > T)	p.Ala40Val (c.119C > T)	compound heterozygous
infantile	p.Arg71Cys (c.211C > T)	p.Glu191Lys (c.571G > A)	compound heterozygous
infantile	p.Arg71Cys (c.211C > T)	p.Glu191Lys (c.571G > A)	compound heterozygous
infantile	p.Arg71His (c.212G > A)	p.Glu191Lys (c.571G > A)	compound heterozygous
infantile	p.Arg71His (c.212G > A)	p.Glu191Lys (c.571G > A)	compound heterozygous
infantile	p.Thr100Met (c.299C > T)	p.Glu191Lys (c.571G > A)	compound heterozygous
infantile	p.Thr141Ile (c.422C > T)	p.Arg428Pro (c.1283G > C)	compound heterozygous
infantile	p.Ala176Thr (c.526G > A)	p.Gly221Arg (c.661G > C)	compound heterozygous
infantile	p.Ala176Thr (c.526G > A)	p.Arg391Valfs (c.1117del)	compound heterozygous
infantile	p.Ala176Thr (c.526G > A)	p.Gly334Asp (c.1001G > A)	compound heterozygous
infantile	p.Ala177Thr (c.529G > A)	p.Arg223Trp (c.667C > T)	compound heterozygous
infantile	p.Glu191Lys (c.571G > A)	p.Gly334Asp (c.1001G > A)	compound heterozygous
infantile	p.Glu191Lys (c.571G > A)	p.Gly334Asp (c.1001G > A)	compound heterozygous
infantile	p.Glu191Lys (c.571G > A)	p.Gly456Arg (c.1366G > A)	compound heterozygous
infantile	p.Arg223Trp (c.667C > T)	p.Gly249Val (c. 746G > T)	compound heterozygous
infantile	c.793-14_33del	c.793-14_33del	homozygous
infantile	p.Arg391His (c.1172G > A)		heterozygous
childhood	p.Thre68Met (c.203C > T)	p.Ala177Thr (c.529G > A)	compound heterozygous
childhood	p.Thre68Met (c.203C > T)	p.Glu191Lys (c.571G > A)	compound heterozygous
childhood	p.Tyr117Cys (c.350A > G)	p.Tyr236* (c.708 T > G)	compound heterozygous
childhood	p.Ala176Thr (c.526G > A)	p.Arg428* (c.1282C > T)	compound heterozygous
childhood	p.Ala177Thr (c.529G > A)	p.Leu275Pro (c.824 T > C)	compound heterozygous
childhood	p.Glu191Lys (c.571G > A)	p.Phe328del (c.984-986delCTT)	compound heterozygous
childhood	p.Glu191Lys (c.571G > A)	p.Gly334Asp (c.1001G > A)	compound heterozygous
childhood	p.Glu191Lys (c.571G > A)	p.Gly334Asp (c.1001G > A)	compound heterozygous
childhood	p.Glu191Lys (c.571G > A)	p.Gly334Asp (c.1001G > A)	compound heterozygous
childhood	p.Glu191Lys (c.571G > A)	p.Gly334Asp (c.1001G > A)	compound heterozygous
childhood	p.Glu191Lys (c.571G > A)	p.Gly334Asp (c.1001G > A)	compound heterozygous
childhood	p.Glu191Lys (c.571G > A)	p.Ala377Val (c.1130C > T)	compound heterozygous
childhood	p.Glu191Lys (c.571G > A)	p.Ala377Val (c.1130C > T)	compound heterozygous
childhood	p.Arg136His (c.407G > A)		heterozygous
childhood	p.Arg136His (c.407G > A)		heterozygous
childhood	p.Pro292Thr (c.874C > A)		heterozygous
childhood	p.Pro292Thr (c.874C > A)		heterozygous
childhood	p.Arg71Ser (c.211C > A)		heterozygous
childhood	p.Ala111Thr (c.331G > A)		heterozygous
childhood	p.Glu191Lys (c.571G > A)		heterozygous
childhood	p.Arg272Cys (c.817C > T)		heterozygous
childhood	p.Met295Thr (c.884 T > C))		heterozygous
childhood	p.Arg391Cys (c.1171C > T)		heterozygous
childhood	p.Arg391Cys (c.1171C > T)		heterozygous
childhood	p.Arg391Cys (c.1171C > T)		heterozygous
childhood	p.Asn417Ser (c.1250A > G)		heterozygous
childhood	p.Arg71Ser (c.211C > A)		heterozygous
childhood	n.k.	n.k.	n.k.
childhood	n.k.	n.k.	n.k.

*n.k.* not known

The most common mutation in our cohort is p.Glu191Lys, which was found in 18/48 patients and p.Gly334Asp in 8 patients. Seven patients share the combination of these two frequent ALPL mutations (p.Glu191Lys/p.Gly334Asp, compound heterozygous) but differ in their clinical presentation (2 infantile HPP of whom one needed ventilation and both were treated with asfotase alfa; 5 childhood HPP, only one severe childhood and received asfotase alfa [21]).

### Core diagnostic findings leading to HPP diagnosis

At the time of established HPP diagnosis the following supportive diagnostic findings have been documented: 96% (48/50) showed a documented low AP activity, 88% (44/50) typical HPP-associated symptoms, 58% (29/50) elevated levels of AP substrates (PLP and PEA), 48% (24/50) radiological abnormalities, 18% (9/50) genetic testing of the ALPL gene as performed at the time of diagnosis and in 12% (6/50) an HPP positive family history was known.

### Medication and treatment history

None of our patients received bisphosphonates or PTH analogues. Two patients were treated with growth hormone. One girl was treated for 6 months before making the diagnosis and stopped afterwards [22] and the other one started treatment after making the diagnosis of mild HPP (heterozygous) and small for gestational age constellation without catch up growth. 54% (27/50) received vitamin D supplementation (dose range 500 to 1000 IU per day), 48% (24/50) NSAIDs for pain management and treatment of inflammation (ibuprofen, naproxen), 16% (8/50) phosphate binding agents and 4 patients pyridoxine/vitamin B6 due to cerebral seizures. Half of the patients diagnosed with craniosynostosis (12/24) developed raised intracranial pressure with need for neurosurgical interventions (skull remodelling). Fractures, if evident, could be conservatively treated, in none of the cases surgery was required. Physiotherapy and/or occupational therapy were/was recommended for almost all patients. Seven patients (3 perinatal, 3 infantile 1 childhood) needed mechanical ventilation; 3 patients experienced resuscitation during course of the disease.

At the time of assessment 20/50 patients were receiving AA: 4/4 of perinatal HPP, 11/17 of infantile HPP, 3/29 of childhood HPP (all were homozygous or compound heterozygous). In 14 patients asfotase alfa was started before the European approval 2015 within a phase 2 clinical trial (ENB010–10, ClinicalTrials.govNCT01176266, [15]).

## Discussion

The aim of this investigation was to better understand the epidemiology and clinical course of disease in pediatric HPP in order to improve and shorten diagnostics. Here we report characteristics, medical history, laboratory findings including genotypes and treatment history of 50 pediatric patients with HPP that have been followed at the University Children’s Hospital of Wuerzburg over the last 25 years.

### Cohort

In our cohort more than half of the patients had a so called childhood form. 34% had an infantile form and only 4 out of 50 patients were diagnosed as a perinatal form. Two of the latter died at the age of 3 months. No patient was documented as Odonto HPP. Compared to other reported cohorts our patients seem to be more severely affected which may be probably due to the fact that our center was a study site in clinical studies of AA.

### Diagnostic delay

There was no delay in making the diagnosis in perinatal HPP probably due to the severity of the disease in this small cohort with all of them being treated in a neonatal intensive care ward. Substantial diagnostic delays between median age at first clinical symptoms and age at diagnosis of HPP was noted in infantile (12 months) and even prolonged in childhood HPP (22.5 months). Unfortunately more differentiated information is lacking: number of doctor contacts, number of diagnostic procedures (laboratory or imaging), exact time from the first clinical symptom until referral to a center, e.g.. Similar findings have been published by Högler et al. with a diagnostic delay of 20.4 months in children (*n* = 90) and 47.5 months in adults (*n* = 52) documented in the Global HPP Registry [23]. There may be several reasons for the diagnostic delay, including low awareness, heterogeneity of disease manifestations especially in clinically “milder” forms and lack of diagnostic guidelines.

### Medical history

Medical history documented systemic manifestations of HPP in perinatal, infantile and childhood HPP, generally consistent with HPP-related clinical symptoms described in the literature [7–9]. First results of the Global HPP Registry documented the importance of considering nonskeletal manifestations as part of the diagnosis of HPP in children and adolescents without differing between age groups [23]..

Interestingly, the most frequent first symptom in our cohort was failure to thrive (34%). Therefor testing for AP activity should be part of a routine diagnostic workup in infants and children with problems in gaining weight, growth retardation and especially with additional musculoskeletal problems (of course among other blood parameters and further diagnostic work up according to local and international guidelines). While suggesting this, one has to keep in mind, that children with feeding and growth disorders due to other means than HPP, often have reduced AP levels due to general lack of nutrients, including zink as the catalytic ion of TNAP. As a consequence, careful analysis of AP levels and, in case of suspiciously low levels, subsequent diagnostic measures including the analysis of TNAP levels in leukocytes, substrate analysis and genetic analysis may have to be considered.

Over time impairment of motor skills, impairment of mineralization, premature loss of teeth and failure to thrive were most commonly reported in our cohort. Impairment of mineralisation in infantile and childhood HPP may also be underestimated due to the necessity of radiation exposure. In our cohort we were reluctant to use X-ray diagnostics as they were not performed routinely just in case of acute or prolonged pain problems, suspicion of fractures, e.g. ultrasound and MRI diagnostics were used instead [24]. Findings reported in a natural history study of 101 affected children published by Whyte et al. support the clinical impression that the natural history of this disorder is typically one of a chronic but stable condition [25].

Our data also support the fact that HPP in children, regardless of subtype, is not characterized by genuine pathologic fractures, excluding the generalized mineralization deficit in the perinatal form. Other pediatric bone diseases such as osteogenesis imperfect are prone to recurrent or poor healing fractures [26]. In contrast, adult HPP patiens may well exhibit a significant fracture risk [12]. Musculoskeletal pain has been documented for almost half of the patients being aware that it is difficult to diagnose in small infants probably resulting in an underestimation at least in this age group. On the other hand one cannot completely exclude the possibility of chronically developing myopathies caused by chronically elevated proinflammatory substrate accumulation, which might contribute to explain the higher incidence (55%) of such conditions in the group of childhood HPP, where patients suffered already for some years from this inborn error of metabolism. We found, that in childhood HPP musculoskeletal pain may be the leading symptom and may also be one cause of diagnostic delay if not adequately assigned. In general it appears that a (not exactly quantifiable) threshold of minimum requirement AP activity exists, beyond which important facets of tissue formation and maturation are impaired concerning e.g. the CNS, the lung and bone mineralization, while in milder forms of the disease accumulating substrates can still produce chronic symptoms of dysfunction such as musculoskeletal pain.

Dental manifestations especially premature loss of teeth are common HPP-related symptoms that can be found in all ages and should always prompt a dentist to advise patients and their families for further diagnostic procedures including AP testing [12, 17, 25].

**Fig. 1:**
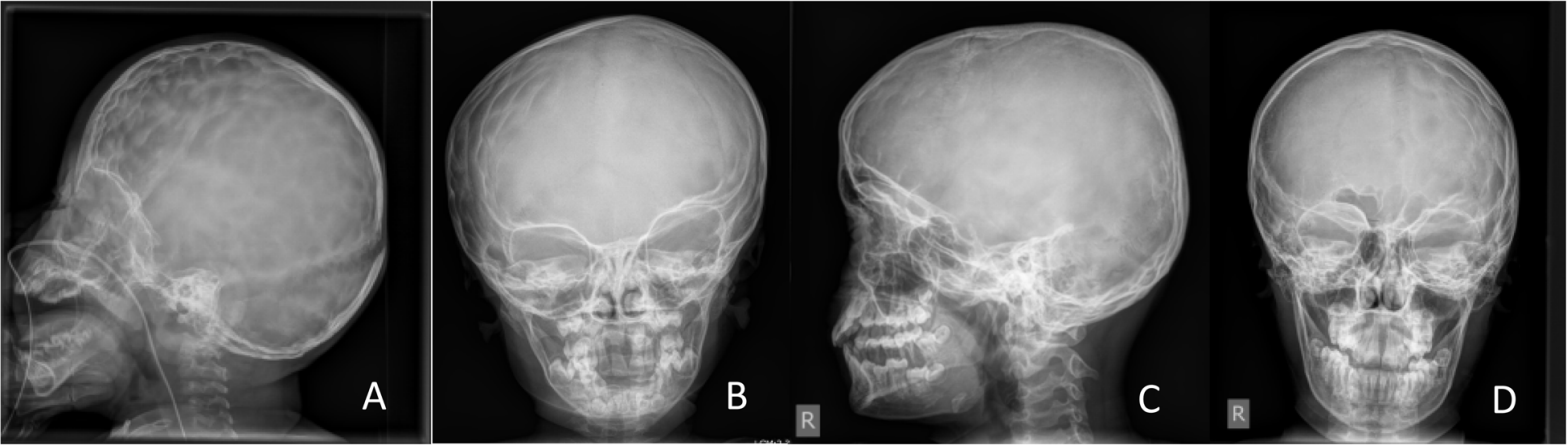
Craniosynostosis in HPP: **a:** Lateral skull X-ray of a 2 year old girl with premature fusion of both coronal sutures and signs of raised intracranial pressure, copper beaten skull. **b:** A.p. skull X-ray of a 5 year old girl with premature fusion of the left cornal suture with signs of raised intracranial pressure – impressiones temporal, right side. **c** and **d:** lateral and a.p. skull X-ray of a 12 year old boy with premature fusion of the left cornal suture and impressiones digitatae predominant on the left side

HPP is associated with an increased incidence of craniosynostosis, hydrocephalus, syringomyelia and chiari malformation [27]. In our cohort craniosynostosis has been diagnosed in almost half of the patients predominantly in perinatal and infantile forms. This percentage is much higher than in other cohorts which might be due to the fact that patients were seen in a multidisciplinary team including an experienced pediatric neurosurgeon. Half of them developed raised intracranial pressure (diagnosed by cranial X-rays, eye examination - papilledema, lumbar punction with measurement of cerebospinal fluid opening pressure) and followed by neurosurgical intervention (skull remodelling). This highlights the importance to carefully look for craniosynostosis in patients with pediatric HPP in particular in more severely affected individuals with early disease manifestations and to follow them very closely in an experienced center for pediatric neurosurgery. The more sutures fuse prematurely the more likely it results in raised pressure due to imbalance between the space requirement of the growing brain and the limited intracranial space [26]. This has also been found in our cohort of HPP patients. But interestingly radiological signs of raised intracranial pressure can also be observed in patients with only one single suture being involved; e. g. in a girl (infantile HPP) with premature fusion of the left coronal suture (Fig. 1). This girl did not show clinical symptoms of elevated pressure, no papilledema and an unremarkable MRI without chiari malformation or syringomyelia.

As craniosynostosis usually is a very slow and chronic process it mostly does not result in obvious acute neurological symptoms based on elevated intracranial pressure, such as headache, nausea, vomiting or altered consciousness. It must also be taken into account that after a neurosurgical therapy the affected suture may fuse again prematurely. The reason for rapid desmal reossification or sclerosis is not understood. In addition, premature closing of the sutures may come along with ossification/calcification of the meningeal membranes, a particular aggravation of the complexity of the neurosurgical procedure. In our cohort 3 patients needed a second operation. Therefore, patients should be monitored for intracranial pressure at least until the end of brain/skull growth which is around 12 years of age. As the reported incidence of craniosynostosis and even craniosystenosis in pediatric HPP seems to be remarkably lower [15–19] this fact adds an important feature to the protocol/algorithm that we have to be aware of in diagnosis and guidance of these patients.

### Laboratory findings and genotypes

AP activity is almost absent in our perinatal forms, very low in infantile HPP (30, reference level 110–590 U/l) and low in childhood HPP (45, reference level 110–550 U/l). From the literature it is known for pediatric HPP that AP levels seem to correlate with age of onset and negatively correlate with disease severity [7–9].

In two HPP-patients with a positive family history AP measurement was in the lower normal range at time of diagnosis (both heterozygous genotype). Both showed suggestive symptoms during the course of the disease and AP activity fell below the lower limit later on. These findings highlight the need for careful clinical assessment, which includes measurement of AP activity also over time (several times if applicable) in children with a positive family history or/and HPP related symptoms. Especially heterozygous carriers harbouring a dominant negative mutation may show a very mild phenotype. Making the diagnosis in these individuals is a great challenge. The “carrier” status of a single mutation should eventually be clinically unremarkable, almost. The physician has to be aware of the lower limits of age-, and sex adjusted AP reference intervals and clinical conditions also resulting in reduced levels.

PLP values were increased in our infantile and childhood patients and may be helpful in establishing the diagnosis of HPP especially if clinical findings and AP measurements are somehow not clear or borderline. One must also be aware that PLP measurement may not be helpful in case of vitamin B6 or multivitamin intake (treatment of seizures, dietary supplement e.g.).

Our findings support the importance to check calcium phosphate metabolism in all patients in general over time (with or without ERT). Especially in more severe forms a low calcium diet may be necessary, at least before ERT. During ERT calcium levels tend to go down, presumably caused by an uptake into bone (“hungry bones”) and need to be monitored even more closely.

In our cohort for all but two patients results of genetic testing were available and a high number of different mutations could be found. Most of our patients had a compound heterozygous genotype, 2 had a homozygous genotype. All patients with a single heterozygous mutation (two of them are known to have a dominant negative effect) were not severely affected. Interestingly p.Glu191Lys has been found in 18, p.Gly334Asp in 8 and the combination of both most common mutations in 7 patients (2 infantile, 5 childhood). P.Glu191Lys is known to occur with a high frequency (up to 55%) in HPP patients with European ancestry and revealed a moderately reduced AP activity in our in vitro testing (68% wild type AP enzyme activity) and it has no dominant-negative effect [21]. P.Gly334Asp has been found previously in homozygous Mennonite patients (founder effect) and in vitro testing showed very low residual activity (1.2%) of this severe mutation and a clear dominant negative effect [21].

The very high number of different mutations in the ALPL gene with various effects on the enzymatic activity in in vitro studies has been correlated with the high variability of phenotypes observed in patients with HPP [28]. According to our clinical observations, however, intra- and interfamilial variability of phenotypes can also be observed in patients with identical genotypes suggesting that additional genetic confounders, as well as epigenetic or environmental factors, may also be involved individually or even at a tissue level [21].

### Diagnosis of the disease

In our cohort in most cases typical HPP-associated symptoms and subsequently a low AP activity level led to the diagnosis of HPP. To a minor extend high substrate levels were available at time of diagnosis in addition. Substrate analysis was performed only in AP borderline cases or within a clinical trial. Furthermore, a positive family history may lead to further diagnostic procedures. Radiological abnormalities at the time of diagnosis were only documented in less than half of our patients. This may be due to restrictive usage of X-rays with regard to radiation exposure for children. X-ray diagnostics were performed in case of suspicion of fracture or elevated intracranial pressure. Currently X-rays are performed more often due to low exposure doses in modern X-ray equipments and to assess the individual bone phenotype in the light of an approved bone-targeted therapy.

It still remains a challenge to establish diagnosis of HPP in heterozygous carriers who might present with non-typical or mild symptoms, nevertheless of relevance for the patient (“carrier” status versus HPP patient, as discussed previously).

### Treatment of pediatric HPP

In our cohort nobody was treated with bisphosphonates as these drugs should be avoided because of their similarity with inorganic pyrophosphate and because they limit bone turnover resulting in reduced activity levels of bone-specific alkaline phosphatase. Supplementation with vitamin D seems reasonable due to low serum levels (Table 3). Treatment with NSAIDs was performed in almost half of our patients mainly during childhood when musculoskeletal pain and /or inflammation has been documented (on demand or 4 to 8 weeks).

The high percentage of patients receiving Asfotase alfa (AA) may be explained as our center was a study site in clinical studies of AA before the approval by the EMEA. All patients treated with AA showed compound heterozygous or homozygous mutations in the ALPL gene. All benefited from treatment with regard to bone, muscle, growth, e.g. Further treatment outcome details will be reported elsewhere.

## Conclusion

Reported findings support our clinical impression of HPP as a chronic musculoskeletal disease with multi-systemic manifestations. Diagnosis is often delayed in particular in children with not life-threatening disease manifestations such as impaired motor skills, ricket-like changes, musculoskeletal pain and inflammation for example.

Our natural history information provides detailed insights into the prevalence of different symptoms, which can help to improve and to shorten the time to diagnosis. As pediatric HPP may mimic rheumatologic conditions one has to keep in mind that simple laboratory tests (AP activity in the serum, PLP in the plasma) can substantiate the clinical differential diagnosis. Genetic testing for mutations in the ALPL gene may add additional information for making the diagnosis of HPP.

The very mild end of the spectrum of even unspecific symptoms poses a particular problem, not to “overdiagnose” HPP. Especially in case of mild or unspecific complaints and a moderately reduced AP, diagnosis should be evaluated very carefully if only one single mutation is found in the ALPL gene. Promising therapeutic options such as ERT might help to optimise medical care with regard to the bone phenotype when an early diagnosis is made.

## Data Availability

The datasets used and/or analysed during the current study are available from the corresponding author on reasonable request.

## Abbreviations

AA: Asfotase alfa
HPP: Hypophosphatasia
PEA: Phosphoethanolamine
PL: Pyridoxal
PLP: Pyridoxal-5′-phosphate
PPi: Inorganic pyrophosphate
TNAP: Tissue-nonspecific alkaline phosphatase
